# Extracellular proteins of *Trametes hirsuta s*t. 072 induced by copper ions and a lignocellulose substrate

**DOI:** 10.1186/s12866-016-0729-0

**Published:** 2016-06-13

**Authors:** Daria V. Vasina, Andrey R. Pavlov, Olga V. Koroleva

**Affiliations:** A.N. Bach Institute of Biochemistry, Research Center of Biotechnology of the Russian Academy of Sciences, 33, bld. 2 Leninsky Ave, Moscow, 119071 Russia

**Keywords:** White-rot fungi, Secretome profiling, Lignolytic enzymes, Lignocellulose degradation

## Abstract

**Background:**

Fungi are organisms with the highest natural capacity to degrade lignocellulose substrates, which is enabled by complex systems of extracellular enzymes, whose expression and secretion depend on the characteristics of substrates and the environment.

**Results:**

This study reports a secretome analysis for white-rot basidiomycete *Trametes hirsuta* cultivated on a synthetic media and a lignocellulose substrate. We demonstrate that *T. hirsuta* st. 072 produces multiple extracellular ligninolytic, cellulolytic, hemicellulolytic, peroxide generating, and proteolytic enzymes, as well as cerato-platanins. In contrast to other white rot species described earlier, which mostly secreted glucanases and mannosidases in response to the presence of the lignocellulose substrate, *T. hirsuta* expressed a spectrum of extracellular cellulolytic enzymes containing predominantly cellobiases and xylanases. As proteomic analysis could not detect lignin peroxidase (LiP) among the secreted lignin degrading enzymes, we attributed the observed extracellular LiP - like activity to the expressed versatile peroxidase (VP). An accessory enzyme, glyoxal oxidase, was found among the proteins secreted in the media during submerged cultivation of *T. hirsuta* both in the presence and in the absence of copper. However, aryl-alcohol oxidase (AAO) was not identified, despite the presence of AAO enzymatic activity secreted by the fungus.

The spectra of the expressed enzymes dramatically changed depending on the growth conditions. Transfer from submerged cultivation to surface cultivation with the lignocellulose substrate switched off expression of exo-β-1,3-glucanase and α-amylase and turned on secretion of endo-β-1,3-glucanase and a range of glycosidases. In addition, an aspartic peptidase started being expressed instead of family S53 protease. For the first time, we report production of cerato-platanin proteins by *Trametes* species. The secretion of cerato-platanins was observed only in response to contact with lignocellulose, thus indicating a specific role of these proteins in degradation of the lignocellulose substrates.

**Conclusions:**

Our results suggest a sequential mechanism of natural substrate degradation by *T. hirsuta*, in which the fungus produces different sets of enzymes to digest all main components of the substrate during cultivation.

**Electronic supplementary material:**

The online version of this article (doi:10.1186/s12866-016-0729-0) contains supplementary material, which is available to authorized users.

## Background

Lignocellulose is a major structural component of plants, which is considered the most abundant substance in biomass available on Earth [1]. It is an attractive and renewable natural resource for production of energy and a range of important substances for modern technologies. Bioconversion of lignocellulose materials is a key issue of the biotechnological applications, as a major component of lignocellulose, lignin, has very high resistance to chemical and biological degradation due to complex three-dimensional structures of its phenylpropanoid units [2] and prevents access to the cellulose constituents. A possible approach to solve this problem suggests use of extracellular oxidative enzymes (oxidoreductases) secreted by lignin degrading fungi, as they play important role in degradation of plant cell wall, containing the lignocellulose components during the fungal growth [3, 4]. The complex of extracellular lignin-modifying enzymes (LMEs), H_2_O_2_-generating enzymes, organic acids, and other incompletely characterized components compose the lignin-modifying system (LMS) of an organism, which defines specific pathways and efficiency of the natural substrate degradation.

Currently, many enzymes of wood-degrading fungal complexes are identified and isolated, but the role of individual enzymes in lignocellulose degradation is not described sufficiently. Only a small number of secreted proteins have been characterized, although it is clear that the fungal multicomponent enzymes systems are much more complicated, and the full spectrum of effectors involved in the degradation and modification of lignocellulose remains unknown. Also, genome sequencing of new plant degrading basidiomycetes found new enzymes related to plant polysaccharide degradation suggesting new roles for previously unknown enzymes. Recently, several new families were added to the CAZy database and characterized in multiple research. In addition, new functions for already characterized enzymes are reported, such as the ability of lytic polysaccharide monooxygenase (LPMO) to cleave not only cellulose, but also hemicelluloses [5].

**Fig. 1:**
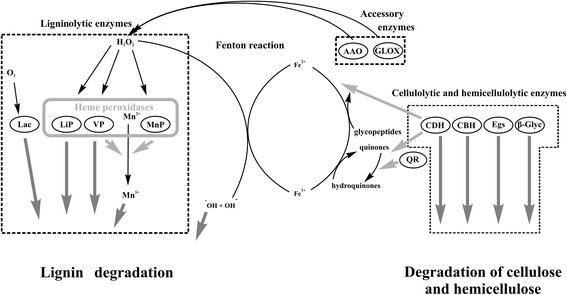
Degradation of lignocellulose by basidiomycetous white-rot fungi. Schematic presentation of the major steps and essential enzymes. Lac - laccase; LiP – lignin peroxidase; VP - versatile peroxidase (Mn^2+^- independent peroxidase); MnP – manganese peroxidase; AAO - aryl-alcohol oxidase; GLOX - glyoxal oxidase; QR - NADH-quinone reductase; CDH - cellobiose dehydrogenase; CBH - cellobiohydrolases; EGs - endogluconases; β-Glyc - β-glycosidases (glycoside hydrolases). Dark grey arrows show reactions which directly modify components of lignocellulose. Reactions producing intermediate substances are shown by light grey arrows or thin black arrows

The degradation pathways of natural substrates by individual fungi (Fig. 1) are determined by the profiles of extracellular enzymes [6]. It is important that the efficiency of degradation is controlled both by composition and the relative amounts of the components of the LMS [7, 8]. There could be significant differences between the proteins expressed by white rot fungi and brown rot fungi grown in identical media. Hori et al. [9] compared secreted protein profiles of white rot fungi *Bjerkandera adusta* strain HHB-12826-SP, *Ganoderma* sp. strain 10597 SS1, *Phlebia brevispora* strain HHB-7030, *Dichomitus squalens*, and *Trametes versicolor* with those of brown rot fungi *Fomitopsis pinicola* and *Wolfiporia cocos,* when they grew using ground aspen as sole carbon source, and found that glycoside hydrolases GH6, GH7, LPMO (GH61), and cellobiose dehydrogenase (CDH) were secreted only by the white rot fungi. However, only the brown rot fungi produced quinone reductase. Also, the expression profiles of the enzymes that are responsible for wood decay were essentially different for white rot fungus *Phanerochaete Chrysosporium* and for brown rot fungus *Postia placenta* [10, 11]. The comparison of the transcriptomes and the secretomes of these fungi both demonstrated different profiles of the expressed genes and the secreted proteins, suggesting complex regulation of the lignin-degrading system involving all levels of biosynthesis of the enzymes.

It is known that most active lignin degradation can be carried out by white rot fungi, primary saprotrophes, which are capable of “mineralizing” lignins. Therefore, for this study we have chosen *Trametes hirsuta* sp. 072 – an efficient lignin degrader producing high level of laccase and peroxidase in various media, especially when supplied with specific inducers, phenolic substances or lignocellulose materials [12–14]. Also, as virtually no information was published about the effect of copper ions (the inducers of laccase expression) on fungal secretome profile during lignocellulose degradation, we analyzed the enzymatic activity and the time-dependent secretomes of *T. hirsuta* in lignocellulose-containing media both in the presence and in the absence of Cu^2+^.

When mechanisms of lignocellulose degradation are explored, cereal straws are often used as fungal substrates [15–18]. As large amount of cereal straws are produced by agriculture, utilization of the waste straw is an important problem due to distinct resistance of straw to microbial degradation, as it contains significant amount of lignin [19] and has low content of nutrients, especially nitrogen [20]. Hence, understanding straw degradation by fungal strains could bring about new approaches of straw utilization and use in various biotechnologies. Therefore, in this study we used ground oat straw, as this substrate allows for fast release of products that form during decay of the lignocellulose and can induce expression of specific proteins.

The main goal of this study was monitoring enzymatic activity and protein secretion during fungal growth under controlled conditions in a synthetic medium and in the presence lignocellulose substrate. Also, the fungus was cultivated under the same conditions with added CuSO_4_ to monitor the response of the laccases’ biosynthesis to copper ions. Using this approach, it was possible to examine changes in the profile of secreted proteins, depending on the conditions of the fungus cultivation.

## Methods

### Cultivation of the fungal strain

Mycelial cultures of Basidiomycete *Trametes hirsuta* 072 used in this study were provided by the Collection of the Komarov Botanical Institute, Russian Academy of Sciences (St. Petersburg, Russia).

The mycelium was precultivated at 26–28 °C in 750-ml Erlenmeyer flasks containing 200 ml of Glucose Peptone (GP) medium (3.0 g peptone, 10.0 g glucose, 0.6 g KH_2_PO_4_, 0.4 g K_2_HPO_4_, 0.5 g MgSO_4_, 50 mg MnSO_4_, 1 mg ZnSO_4_, and 0.5 mg FeSO_4_ per 1 l of water; pH 6.0) and ceramic beads [21]. Then the mycelium was disrupted into small fragments by ceramic beads and used to inoculate either submerged cultivation of *T. hirsuta* 072 in GP medium on a rotary shaker at 160 rpm or static surface cultivation (without shaking) of the fungus in the presence of lignocellulose (LC medium; 50 g/l of milled oat straw d ≥ 1 mm in GP medium separated from the fungus by a nylon mesh [18]. The average content of copper in the oat straw was 3.6 microgram per gram dry matter, as determined by inductively coupled plasma mass spectrometry (ICP-MS)) at the same temperatures and volumes as during the precultivation. To study the effect of copper on laccase biosynthesis, both GP and LC cultivation media were supplied with 1 mM of CuSO_4_. In a separate experiment, static cultivation in GP medium without inducers was used to assess specific effects of LC and submerged cultivation on production of extracellular enzymes. To determine the dry weight of biomass, the fungus was cultivated at the specified conditions in individual 750-ml Erlenmeyer flasks for the defined time, harvested, and dried at 105 °C. The weights were recorded when they achieved constant values after drying. Cultural liquids were used for secretome analysis.

### Determination of protein concentration

The samples of the cultural broth were centrifuged for 5 min at 9000 g and Protein concentrations in the supernatants were determined by the method of Bradford [22] according to the reagent producer’s instructions (Pierce, USA). All measurements were performed in triplicate.

### Enzyme activity assays

Laccase activity was measured by decrease of absorbance at 520 nm using syringaldazine (Sigma, USA; ε_520_ = 65,000 M^−1^ · cm^−1^) as a chromogenic substrate. The enzymatic reaction was carried out for 3 min in 2 ml reaction mixtures containing 0.1 M sodium acetate buffer, pH 4.5, 0.42 mM syringaldazine, and the required amount of the cultural liquids to determine initial rates of the substrate oxidation [23].

Manganese peroxidase (MnP) activity was determined directly by formation of Mn^3+^- tartrate complex (ε_238_ = 6500 M^−1^cm^−1^) during oxidation of 0.1 mM MnSO_4_. 2 ml reaction mixtures contained 0.1 mM MnSO_4_ in 0.1 M sodium tartrate, pH 5.0 and 0.1 mM H_2_O_2_. The initial rates of reaction were measured by recording linear increase of absorbance at 238 nm for 3 min [24].

Mn^2+^- independent peroxidase (versatile peroxidase, VP) activity was evaluated by formation of the veratraldehyde (ε_310_ = 9300 M^−1^cm^−1^) from veratryl alcohol. The enzymatic reaction was assayed in 2 ml mixtures containing 2 mM veratryl alcohol, 0.1 M sodium tartrate, and 0.1 mM H_2_O_2_, and culture supernatant at pH 3.0, and the change of optical density was recorded at 310 nm for 3 min.

To measure lignin peroxidase (LiP) activity, we used similar conditions of the reaction, but veratryl alcohol concentration was set to 8 mM, and reaction was carried out at pH 4.5. [24, 25].

For evaluation of Aryl-alcohol oxidase (AAO) activity, the above reaction conditions were modified to measure formation of veratraldehyde from 5 mM veratry1 alcohol in 0.1 M sodium phosphate, pH 6.0. [26].

All measurements of enzymatic activities were carried out at +25 °C in the thermostated cell of LAMBDA 25 UV/Vis System spectrophotometer (PerkinElmer, USA). One unit (U) of enzyme activity was defined as the amount of an enzyme that transformed 1 μmol of the specific substrate under assay conditions. The background reactions were measured in the corresponding cultural media which did not contain the fungus. All assays were performed in triplicate.

### Secretome extraction

Samples of cultural broth were collected at specified times and filtered through syringe filters (0.45 μm, Sartorius, Germany) to remove small particles. Then, mycelium-free culture broth was concentrated 10-fold and simultaneously desalted by tangential flow ultrafiltration system Pellicon® XL with a 5 kDa MW cut-off membrane Biomax 5 (Millipore, USA). Then the proteins were precipitated using one of the following methods:

*Chloroform/methanol precipitation* was performed as described by Wessel and Flügge [27]. To 1 volume (20 ml) of the concentrated broth were added 4 volumes (80 ml) of methanol, 1 volume (20 ml) of chloroform, and 3 volumes (60 ml) of H_2_O, mixed and incubated for 5 min at 4 °C. After centrifugation at 9000 g (4 °C, 2 min), the aqueous phase was removed without disturbing the interphase and another 300 ml of methanol were added to the precipitate. The samples were incubated at 4 °C for 5 min, the proteins were pelleted at 2000 g (4 °C, 5 min) and dried on Speed-Vac.

*Modified Chloroform/methanol precipitation* was carried out as described above after additional concentration of the sample by overnight dialysis against a solution containing 30 % of 30 mM K-phosphate buffer, pH 7.5 and 70 % glycerol (v/v) at 4 °C [28].

*Acetone precipitation* was done by mixing ice-cold acetone with the concentrated cultural broth (4:1 v/v), overnight incubation at −20 °C, and centrifugation as above. The precipitate was separated by centrifugation (15 min, 4400 rpm). The pellet was washed once with ice-cold acetone and air-dried [29].

*Acetone-TCA method* included protein precipitation with acetone containing 13.3 % TCA and 0.093 % β-mercaptoethanol (at 1:1 sample/precipitant ratio) for 12 h at −20 °C [30]. The precipitate was separated by centrifugation (15 min, 4400 rpm), washed twice by 0.07 % β-mercaptoethanol in acetone and dried for 5 min in airflow at 20 °C. Then the precipitate was dissolved in 0.05 M Tris–HCl buffer (pH 6.8) and the proteins were re-precipitated using 2D Protein Kit according to manufacturer’s instructions (Bio-Rad, USA).

*2D Protein Kit precipitation* alone was done according to the manufacturer’s instructions.

The proteins were dissolved in resolubilization solution containing 5 % Servalytes (pH 3-10; Serva Electrophoresis, Germany), 1 % DTT, 4 % 3-[(3-cholamidopropyl) dimethyl-ammonio]-1-propanesulfonate (CHAPS), 7 M urea, 2 M thiourea and treated in an ultrasonic bath (Bandelin Sonorex, 35 kHz, 120 W) for 10 min at 20 °C. After the treatment, the reaction mixture was incubated for 1 h at 20 °C and centrifuged for 5 min at 9 000 g. The supernatant was collected and used for further studies.

### Two-dimensional electrophoresis (2DE)

Two-dimensional electrophoresis was done according to O’Farrell [31] on a Protean II xi 2-D Cell system (Bio-Rad). 150–200 μg of the proteins were applied per a tube with Servalytes (pH gradient 3–10), and the isoelectrofocusing was performed using the following conditions: 100 V for 45 min, 200 V for 45 min, 300 V for 45 min, 400 V for 45 min, 500 V for 45 min, 600 V for 45 min, 700 V for 10 h, 900 V for 1.5 h.

After IEF, the samples were incubated at room temperature for 15 min in 0.5 M Tris–HCl buffer (pH 6.8) containing 6 M urea, 2 % SDS, and 10 mM DTT (to prevent oxidation of sulfhydryl groups of the proteins). The subsequent electrophoresis of the samples was performed in a gradient SDS polyacrylamide gel (7.5–25 %) at 300 V. The visualization of protein components in the gels was achieved by staining with AgNO_3_ or, for subsequent mass spectrometry of the protein samples, with Brilliant Blue R Staining Solution (Sigma, USA). Protein mapping was done with Infinity1000/26MX gel-documenting system (Vilber Lourmat, France). Then, the protein maps were analyzed using ImageMaster 2D Platinum v.7 program (GE Healthcare, UK).

### MALDI-TOF/TOF MS analysis

For MS analysis, small (3–4 mm^3^) pieces of the gels with stained proteins of interest were cut out, incubated in 100 μl of 0.1 M NH_4_HCO_3_ containing 40 % acetonitrile at 37 °C for 20 min to remove coomassie brilliant blue, and the proteins were hydrolyzed by incubation at 37 °C with 15 μg/ml solution of modified trypsin (Promega, USA) in 50 mM NH_4_HCO_3_ for 8 h. Then, 0.5 % TFA in 10 % solution of aqueous acetonitrile was added to the samples, and the solution above the gel containing the protein hydrolysate was collected and used for mass spectrometry with 2,5-dihydroxybenzoic acid (Aldrich, USA) as a matrix. Mass spectra were obtained using a Bruker Ultraflex II MALDI TOF/TOF mass spectrometer (Germany) with a reflectron; the accuracy of measured monoisotopic masses upon recalibration by trypsin autolysis peaks was 0.005 % (50 ppm). The fragmentation spectra were obtained using the tandem mode of the device, and the accuracy on measurement of fragmented ions was at least 1 Da.

Mass spectra data were processed using the FlexAnalysis 3.3 program (Bruker Daltonics, Germany). The search for proteins corresponding to MALDI-TOF/TOF MS data was carried out with Mascot Peptide Mass Fingerprint in the fungal subset of NCBI non-redundant protein sequences taking into account possible oxidation of methionine and modification of cysteine residues. The search with combined data of the peptide masses and peptide fragmentation was performed by Biotools 3.2 (Bruker Daltonics). Additionally, sequences of the peptides individually derived from the fragmentation data were analyzed by BLAST NCBI using the fungal subset of the GenBank database.

## Results and discussion

The white rot fungus *T. hirsuta* st. 072, an efficient wood degrader with a strong system of lignolytic enzymes, partially characterized in our previous studies [12, 14, 18, 21], was chosen as a model organism. Until now, no data on the entire fungal secretome had been reported, mainly, because of difficulties of sample preparation for proteomic analysis, and an optimized procedure was developed for analysis of the fungal extracellular proteins which was essential for this study (see Additional file 1 for details).

### Fungal growth and enzymes activity profiles

The growth phases of fungi affect profiles of both secreted and intracellular proteins [32]. Depending on the composition of the nutrient medium, timing of the growth phases can vary. Activities of specific enzymes can be observed at different times, depending on the physiological and biochemical properties of the tested strain, the composition of culture medium, substrate stage degradation, and the presence of biosynthesis inductors. In this case the time of cultivation was chosen based on previous results of laccase production dynamic on different media [21, 33]. The activities of LME were monitored using common target enzymes [34–37]: laccase, peroxidase complex (MnP, LiP, VP), and aryl-alcohol oxidase.

**Fig. 2:**
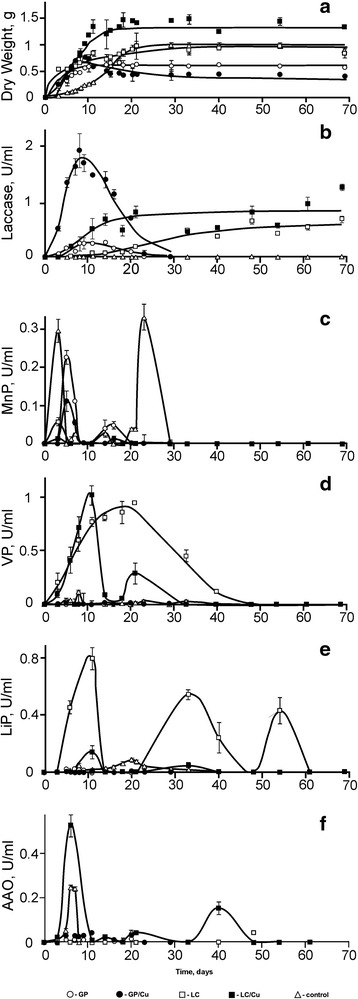
Fungal growth and activity of lignin degrading enzymes of *T. hirsuta* 072. The dependencies on time are shown for the amount biomass and enzymatic activities measured on different days of growth (see *Materials and Methods*). Panel **a** depicts the dependencies for the dry weight, and panels **b**-**f** show the activities of laccase, manganese peroxidase, Mn^2+^- independent peroxidase (versatile peroxidase), lignin peroxidase, and aryl-alcohol oxidase, respectively. The results are shown for the submerged cultivation in GP medium (circles), static surface cultivation in GP medium (triangles), and static surface cultivation in LC medium (squares). Filled symbols indicate cultivation in the presence of 1 mM of CuSO_4_

Figure 2 shows dynamics of biomass growth and activities of lignin degrading enzymes during cultivation of *T. hirsuta* 072 in GP and LC media. The exponential growth phase terminated as early as by 8 to 10 days of cultivation in GP medium with agitation, then the stationary phase (without Cu^2+^) or lysis (with Cu^2+^) could be observed (Fig. 2a). Surface cultivation produced prolonged exponential growth (up to 18–21 days before going to the stationary phase) and higher yields of the biomass both in GP and LC media. In the presence of CuSO_4_, the accumulation of the mycelial biomass in both media was 1.5–1.6-fold higher than without the inducer, but the growth dynamics did not have a significant change.

Panels B-F show profiles of enzymatic activities for extracellular enzyme during cultivation of *T. hirsuta*. The laccase production reached its maximal level on 8^th^ day of cultivation in GP medium with agitation, both in the presence and in the absence of Cu^2+^; however, the addition of CuSO_4_ increased laccase activity up to 8-fold. In contrast, cultivation in GP medium without agitation produced 10-fold lower levels (~0.02 U/ml) of the enzyme activity, as compared to the growth with agitation in the same media (panel B), which did not increase with the increase of the fungal biomass. Cultivation in LC without agitation induced significant laccase activity, which increased with the fungal growth and reached plateaus on the stationary phase of growth both in the presence and in the absence of CuSO_4_. Under these conditions CuSO_4_ did not induce laccase production significantly.

The profiles of MnP activity in GP media showed two major peaks: the first one during the exponential growth and the second one on the stationary phase. Agitation, addition of CuSO_4_, or lignocellulose - all suppressed the production of the active enzyme. As illustrated in Fig. 2c, the first MnP activity peak occurred on the 3^rd^ day of growth without agitation (both in GP and LC) and shifted to the 5^th^ day for the submerged cultivation. The highest suppression was observed in LC, and the effects of lignocellulose and CuSO_4_ did not interfere. However, neither Cu^2+^ nor lignocellulose changed the time of the observable peaks (the second MnP activity peak completely disappeared in LC).

In contrast to MnP, activities of both versatile peroxidase (Panel D) and lignin peroxidase (Panel E) were barely detectable under conditions of submerged cultivation in GP medium. However, the activities produced in GP medium without agitation were also very low. Both LiP and VP activity production were much higher in LC media, suggesting induction of these enzymes by lignocellulose, and suppression of both activities by added CuSO_4_ (strong effect for LiP and at least partial effect for VP) could be observed.

The secretion of peroxide generating aryl-alcohol oxidase demonstrated more complicated behavior. The major peak of activity appeared on 6^th^ day of surface cultivation, during the exponential growth phase. Agitation clearly inhibited the production of the enzymatic activity. The effect of CuSO_4_, however, was the opposite of that observed with the heme peroxidases, as the activity of the enzyme significantly increased. Surprisingly, lignocellulose alone almost abolished the enzyme production until late stationary phase. Nevertheless, addition of CuSO_4_ eliminated the suppression and induced the enzyme production without shifting time of appearance for the major peak (Panel F).

High levels of activity were found for MnP, LiP, VP, as well as for an accessory peroxide-generating enzyme AAO (Fig. 2), using the enzyme activity assays proposed earlier to monitor the levels of the peroxidases and the accessory proteins in the extracellular media [23–26]. However, the described methods for measuring the enzyme activities of LiP, VP, and AAO all use veratryl alcohol as a substrate (at concentrations 8 mM, 2 mM, and 5 mM, respectively), albeit at somewhat different pH of the reaction, and, therefore, all the activities can contribute into the measured rate of veratryl aldehyde formation at the specific assay conditions, especially, as it is not known what concentration of H_2_O_2_ exists in the growth media. Nevertheless, the individual profiles of the enzyme activities under different growth conditions suggested individual expression patterns for each enzyme (or individual isoforms with distinct enzymatic properties) during the fungal growth, e.g., AAO activity increased by addition of Cu^2+^ and was not specifically induced by LC, as opposed to LiP and VP, which were inhibited by copper ions (Fig. 2). Activities of LiP and MnP had multiple peaks during the fungal growth on the LC substrate, assuming a periodic character of their induction by the products of ligninolysis followed by a new cycle of the substrate degradation. It seems possible that some LiP activity went undetected, since despite the common occurrence of genes similar to those encoding LiP in *Trametes* genus [38], the LiP activity often cannot be measured, most likely, due to low stability of the enzyme [39]. Therefore, the analysis of the secreted proteome offered more reliable technic to estimate the expression of the enzyme.

### Analysis of T. hirsuta secretome

In all cases studied previously [28, 40, 41], natural lignocellulose substrates induced different sets of proteins, as compared to the synthetic media, and often larger number of components could be detected. When transcriptional changes were studied during protein induction by four different wood substrates (lodgepole pine, white spruce, balsam fir, and sugar maple) [42] for the fungus *Phanerochaete carnosa*, only quantitative differences were found for all wood species, suggesting the same set of enzyme activities as a response to wood lignocelluloses. The current secretomic research, including the present work, indicates common patterns for protein induction by various types of lignocellulose and tries to assess proteins induced by natural lignocellulose substrates as compared to synthetic medium with various carbon sources [28, 40, 41]. GP media used in this study were previously characterized and optimized for production of laccase by *T. hirsuta* st. 072 [21].

**Fig. 3:**
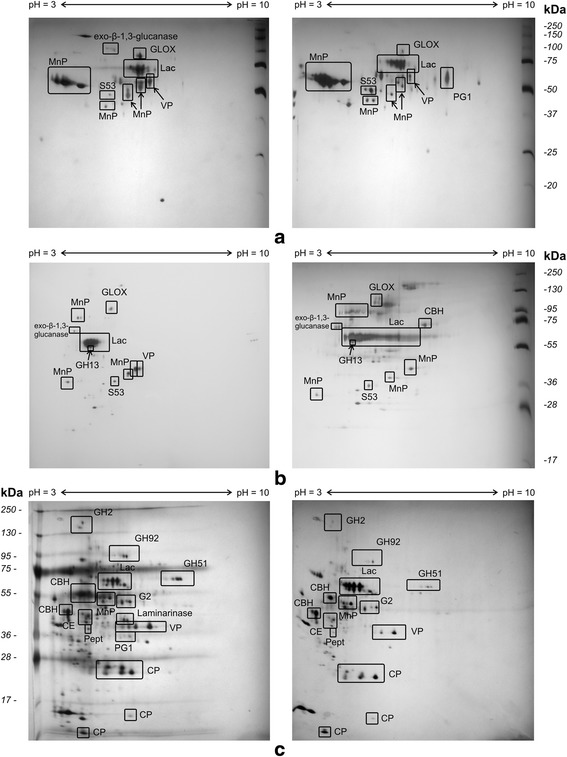
Analysis of *T. hirsuta* 072 secretome. The extracellular proteins were extracted from cultural broth and resolved by 2D electrophoresis (see *Methods*). Proteins secreted in GP medium (submerged growth) without induction (left) and induced by CuSO_4_ (right), respectively are shown on 5^th^ day of cultivation (panel **a**) and on 8^th^ day of cultivation (panel **b**). The extracellular proteins expressed in LC medium (surface growth) are displayed for 3^th^ day of cultivation without and with added CuSO_4_ (panel **c**). To observe differential expression, for each panel, equal amounts of total protein were loaded on both gels. Proteins identified by MALDI-TOF/TOF MS analysis are marked on the gels: Lac – laccase; MnP – manganese peroxidase; VP – versatile peroxidase; GLOX – copper radical oxidase (glyoxal oxidase); PG1 -Endo-polygalacturonase PG1, GH2 – glycoside hydrolase family 2; GH13 – glycoside hydrolase family 13; GH51 – glycoside hydrolase family 51; GH92 – glycoside hydrolase family 92; CBH – cellobiohydrolase I; G2 – glucoamylase G2; CE – carbohydrate esterase family 16; S53 - family S53 protease; Pept - aspartic peptidase; CP - cerato-platanins

For submerged growth in GP media, both without induction and with addition of CuSO_4_, the samples for 2D electrophoresis were collected on 5^th^ and 8^th^ days of cultivation, at the peak of the enzymatic activities in the cultural broth. In addition, a sample collected on the 20^th^ day (the stationary phase/lysis in GP media) was added to the secretome analysis. During surface cultivation in the presence of LC, we analyzed the samples collected on 3^rd^, 11^th^, and 21^st^ days of the fungal growth. Examination of extracellular proteins by two-dimensional electrophoresis showed significant differences in the expression of the protein components depending on media composition and time of cultivation (Fig. 3). As compared to the submerged cultivation in GP media, surface cultivation in the presence of LC drastically increased the number of the expressed proteins. Ascription of the individual protein spots to specific fungal proteins based on MS analysis of the triptic peptides after extraction from the gel found major ligninolytic, cellulolytic, and hemicellulolytic enzymes in the *T. hirsuta* secretomes. The results of protein expression analysis are summarized in Table 1. Additional details on protein identification in Table 1 are summarized in Additional file 2: Table 2s.

**Table 1:** Proteins identified by MALDI TOF/TOF mass spectrometry in *T. hirsuta* 072 secretomes

Identified proteins	Highest Similarity to Proteins (NCBI Accession #, Organism)	Number of Isoforms (Isozymes); -Cu/+Cu						Physiological role
		*GP-medium*			*LC-medium*			
		*5 days*	*8 days*	*20 days*	*3 days*	*11 days*	*21 days*	
*Lignolytic enzymes*								
Laccase^a,b^	KP027478*T. hirsuta*; laccase A^c^	8/13	7/11	7/10	8/8	4/5	5/8	Lignin degradation
Manganese peroxidase^a,b^	JQ388597 *L. gibbosa*; manganese peroxidase 2	10/14	6/4	5/4	4/3	n/a	n/a	
Versatile peroxidase-like^b^	EIW62513 *T. versicolor*; manganese-repressed^d^ peroxidase	2/2	1/0	0/0	4/2	6/4	0/2	
*Glycolytic enzymes*								
Cellobiohydrolase I^b^	EIW64126 *T. versicolor*; cellobiohydrolaseI^e^	n/a	0/4	3/4	4/3	4/2	1/2	Cellulolytic enzymes
Exo-β-1,3-glucanase^b^	EIW63632 *T. versicolor*; exo-beta-1,3-glucanase^f^	2/0	2/3	0/3	n/a	n/a	n/a	Cellulolytic enzymes
Endo-β-1,3-glucanase (Laminarinase)^b^	EIW53084 *T. versicolor*; laminarinase^g^	n/a	n/a	n/a	2/0	2/0	1/0	Cell wall modification
Glycoside hydrolase family 2 protein^b^	EIW63844 *T. versicolor*; glycoside hydrolase	n/a	n/a	n/a	2/1	1/1	n/a	Glycosidic bond cleavage
Glycoside hydrolase family 13 (α-amylase)^b^	EIW55835 *T. versicolor*; glycoside hydrolase	n/a	1/1	1/1	n/a	n/a	n/a	
Glycoside hydrolase family 51 protein^b^	EIW55650 *T. versicolor*; glycoside hydrolase family 51 protein	n/a	n/a	n/a	9/7	8/6	4/5	Major hemicellulolytic enzymes
Glycoside hydrolase family 92 protein^b^	EIW52130 *T. versicolor*; glycoside hydrolase family 92 protein	n/a	n/a	n/a	3/1	7/8	5/7	Cell wall modification
Endo-polygalacturonase PG1^b^	CDO68615 *T. cinnabarina*; glycoside hydrolase family 28 protein	0/1	n/a	n/a	3/0	3/3	2/2	Pectin-degrading enzymes
Carbohydrate esterase family 16 protein^b^	CDO76317 *T. cinnabarina*; carbohydrate esterase family 16 protein	n/a	n/a	n/a	5/5	0/2	2/2	Pectin-degrading enzymes
Glucoamylase (G2)^b^	EIW63814 *T. versicolor*; Glucoamylase^h^	n/a	n/a	n/a	4/3	2/2	n/a	
*Accessory enzymes*								
Copper radical oxidase (Glyoxal oxidase)^b^	CDO70163 *T. cinnabarina*; Copper radical oxidase	2/1	2/2	2/2	n/a	n/a	n/a	Generation of hydrogen peroxide
*Others*								
Family S53 protease^b^	EIW62828 *T. versicolor*; family S53 protease	1/3	1/1	n/a	n/a	0/1	n/a	
Aspartic peptidase^b^	ETW87129 *H. irregulare*; aspartic peptidase	n/a	n/a	n/a	1/0	0/1	n/a	
Cerato-platanins^b^	EIW62257 *T. versicolor*; cerato-platanin	n/a	n/a	n/a	7/8	5/2	2/3	Phytotoxic proteins

^a^Mascot Peptide Mass Fingerprint

^b^BLAST analysis of MSMS - derived peptide sequences

^c^At 69 days of incubation, laccase C also could be detected

^d^EIW62513.1 sequence analysis suggested versatile peroxidase-like folding of the gene product annotated as manganese-repressed peroxidase. Similar sequences were annotated as class II peroxidase and manganese peroxidases

^e^Similar sequence was annotated as glycoside hydrolase Family 7 protein [*Trametes cinnabarina*]

^f^Similar sequence was annotated as glycoside hydrolase family 55 protein [*Hebeloma cylindrosporum* h7]

^g^Similar sequence was annotated as glycoside hydrolase family 16 protein [*Ceriporiopsis subvermispora* B]

^h^Similar sequence was annotated as glycoside hydrolase family 15 protein [*Phanerochaete carnosa* HHB-10118-sp]; n/a – protein was not found on electrophoregram

#### Production of ligninolytic enzymes

The composition of LME can differ significantly for individual basidiomycetes. *E.g.*, it is reported that *Phanerochaete chrysosporium* does not produce classical laccases [43]; in contrast, *Pleurotus ostreatus* does not have lignin peroxidases [6]. Recent genetic studies have found new families of fungal peroxidases [44], such as aromatic peroxigenases of agaricomycetes (mushroom peroxigenases), dye-decolorizing peroxidases, and heme-thiolate peroxidases, which include distinguished chloroperoxidases from *Caldariomyces fumago* [44–47]. Still, the majority of these genes were never expressed, and exact substrate specificities of the corresponding gene products have yet to be defined.

Laccase is considered a major lignin modifying enzyme for *Trametes* genus [48, 49]. In this case, GP medium demonstrated the predominance of the laccase fraction on the electrophoregrams (Fig. 3 a, b) that persisted throughout the cultivation. Without CuSO_4_ in the media, several protein components corresponding to laccase were identified by mass spectrometry in a compact group MW = 60–70 kDa and pI ~ 4. As a result of induction with copper, the number of protein components identified as laccases increased, and their pI values varied in a significantly greater range. However, during the cultivation, the number of the laccase isoforms decreased in both cases, especially in the medium with CuSO_4_ (from 13 in the early exponential growth phase, to 10 - after the transformation to stationary phase) (Table 1). In contrast, although the number of isoforms expressed without copper in LC medium was the same as in GP medium (8), it did not increase in the presence of Cu^2+^. Also, surface cultivation in the presence of lignocellulose did not show progressive decrease of the number of the isoforms in the medium with copper (Table 1). These results, which suggest interference between aromatic products of lignin degradation and Cu^2+^ in regulation of laccase production are consistent with early finding that CuSO_4_ did not change the expression of laccase both on the RNA or protein levels in *Trametes versicolor* if the fungus was grown in the presence of an aromatic substance 2,5- xylidin. Nevertheless, copper could significantly increase the enzyme activity, evidently, as a result of more efficient incorporation at high concentrations [50]. Comparison of tryptic peptides from the laccase protein spots with gene products of recently sequenced laccase mRNAs from *T. hirsuta* [51] could find matches only to laccase A [GenBank: KP027478]. However, after 21 days of surface cultivation in LC medium laccase C [GenBank: KP027479] could be detected, as well. This would suggest turning on expression of additional isozymes, depending on accumulation of the specific inducers on different degrees of lignin degradation. Earlier, similar results were obtained for *Phlebia radiata* that produced two isozymes of laccase, Lac1 and Lac2 after 5 days and after 13 days of cultivation, respectively, as the major forms [52]. As shown previously [51], both Lac1 of *P. radiata* and LacA of *T. hirsuta* 072 reside to the same similarity cluster A, and Lac1 of *P. radiata* belongs to the similarity cluster B. The predominant LacA of *T. hirsuta* was found in multiple isoforms due to glycosylation in all used medium, but the number of the glycosylated forms increased in the presence of Cu^2+^ (Fig. 3; Table 1), which is consistent with previously observed changes in the glycosylation patterns of 2 laccases from *T. versicolor* (which belong to the similarity functional clusters A and B [51]) with addition of metal ions [53].

Another major ligninolytic enzyme, manganese peroxidase, can be found in many isoforms in GP cultivation medium; however, the major group of about 10 acidic isoforms (pI ~ 3; MW = 50-60 kDa) expresses as early as by 5^th^ day of the fungal growth and then completely disappears by 8^th^ day of cultivation (Fig. 3a, b). The expression of these forms corresponds to the profile of MnP activity during the cultivation (Fig. 2c), suggesting their importance for manganese oxidation by the fungus. Apparently, expression of those isoforms is suppressed by lignocellulose, in agreement with the low MnP activity in the LC medium (Fig. 2c). In contrast, addition of CuSO_4_, which also suppress the enzyme activity, had lesser effects on the protein expression. This is curious, as it was shown that copper ions enhanced activity of manganese peroxidases in *Trametes trogii* [54] and increased transcription of MnP-encoding genes in *Ceriporiopsis subvermispora* [55] when the fungi were grown on artificial media without sources of lignocellulose. Among other heme peroxidases, only versatile peroxidase-like protein with similarity to gene sequences in other fungi annotated either as versatile peroxidases or manganese peroxidases [56], but also resembling genes whose transcription is repressed by manganese [57] could be found. The protein expressed both in GP and in LC media, but demonstrates much higher molecular weight isoforms in GP (>50 kDa) than in LC (~40 kDa) in spite of the presence of peptides with identical masses in the triptic hydrolysate.

As shown in Fig. 3 and Table 1, no LiP expression could be found at all used growth conditions, suggesting that some isoforms of VP could be responsible for the LiP - like activity.

Also, an accessory enzyme, glyoxal oxidase, was found in the samples of cultural broth after *T. hirsuta* submerged cultivation both in the presence and in the absence of copper. The other accessory enzyme, AAO could not be identified by MSMS in the resolved proteins of the secretome despite of the AAO - like activity measured in the media. However, low specificity of the AAO activity assay with veratryl alcohol together with the presence of H_2_O_2_ in the media mentioned above could greatly overestimate the activity of this enzyme. Thus, the lignin LMS of *T. hirsuta* st. 072 primarily relays on the activities of various laccase isoforms and VP.

#### Production of cellulolytic and hemicellulolytic enzymes

The secretome of *T. hirsuta* demonstrated a significant number of diverse cellulolytic and hemicellulolytic enzymes. It was previously shown [6, 9, 58] that white rot fungi could produce cellulases that belong to families GH5, GH6, GH7, and GH9 and contain carbohydrate-binding module family CBM1. Also, the oxidoreductases CDH and LPMO (GH61, lytic polysaccharide monooxygenase) were found mostly in white rot fungi. In addition, expression of xyloglucanase (GH74), endo-xylanase (GH10), β-glucuronidase (GH79), acetyl xylan esterase (CE1), and glucuronoyl methylesterase (CE15) were significantly increased in white rot, as compared to brown rot basidiomycetes.

As can be seen in Table 1 and Fig. 3, among the proteins secreted by *T. hirsuta* 072, we found cellobiohydrolase I (CBH) and endo-polygalacturonase (PG1) secreted in all media, exo-β-1,3-glucanase and glycoside hydrolase of family GH13 (only in GP media), and a large set of LC media - induced glycoside hydrolases: endo-β-1,3-glucanase, carbohydrate esterase family 16, glucoamylase (G2), and glycoside hydrolases of families GH2, GH51, and GH92. Thus, in contrast to already described white rot species, which express proteins with predominantly cellobiase and xylanase activities [6, 9, 58], the enzymes secreted by *T. hirsuta* should have mostly glucanase and mannosidase activities.

Active production of this group of enzymes was observed in the beginning of cultivation on 3^rd^ and 11^th^ days, but was lower by 21^st^ day. As they were not observed in the secretomes produced in GP media, indicating that these enzymes did not have constitutive expression, and thus were most likely encoded by the inducible genes.

Presence of CBH, endo-β-1,3-glucanase, 1-3-beta-glucosidase, and glycoside hydrolases specifically on the initial stages of the fungal growth suggested high cellulolytic potential of the *T. hirsuta* strain [59, 60], even if the fungi of *Trametes* genus are generally considered as primarily lignolytic [61]. Most importantly, *T. hirsuta* demonstrates early expression of cellobiohydrolase I, which is necessary for degradation of native cellulose, as it forms new end groups of the polysaccharide by cleaving cellobiose off the non-reducing ends of cellulose [62].

As shown in the present study, change of the cultivation medium, can switch the production of fungal cellulolytic enzymes between different sets. In the absence of LC, only cellobiohydrolase I, exo-β-1,3-glucanase, α-amylase, and endo-polygalacturonase PG1 could be identified. Among them, exo-β-1,3-glucanase and α-amylase could not be distinguished on LC gels, apparently because of relatively low levels of expression. As a result, the submerged cultivation in GP and the surface cultivation in LC display essentially different spectra of glycolytic enzymes.

When *T. hirsuta* grows in GP medium, the fungus, along with CBH, expressed exo-β-1,3-glucanase, which can cleave di- and tetra- saccharides from the end of glucan chains. With LC media, no exo-β-1,3-glucanase was found; however, we identified endo-β-1,3-glucanase, which likely induces internal breaks in the cellulose chain, and thus disrupting the crystal structure of cellulose and facilitating further degradation of cellulose [63]. There were also some reports about β-1,3-glucanases as cell wall-lytic enzymes that are important for the morphology of basidiomycetous fungi [64]. As for exo-β-1,3-glucanase activity, the function of the enzyme can be substituted by several glycosidases expressed in LC media.

Current research suggests that important mechanisms which regulate biosynthesis of cellulases and hemicellulases in fungi are induction and catabolite repression [65]. For several fungi, induction of cellulase and hemicellulase genes can be carried out by soluble hydrocarbons, such as products of hydrolysis or transglycosylation of cellulose or xylans [5, 66, 67]. The catabolite repression in the presence of easily available carbon sources, such as glucose, can affect genes of hydrolytic enzymes involved in plant cell wall degradation [65].

#### Production of accessory enzymes and other proteins

Fungal secretomes can contain significant amounts of proteases [40], especially when a fungus grows on a complex substrate. The content of peptidases and proteinases in such cases can be as high as 20 % of total secretome [68]. Nevertheless, the role of proteases in the decay of lignocellulosic substrates is often underestimated. It is suggested that the secretion of proteolytic enzymes is regulated by the carbon and nitrogen sources ratio and activates at low levels of accessible nitrogen containing compounds [41]. Therefore, in our experiments in nitrogen - rich GP media low expression of the protease pool was expected. Indeed, only one proteolytic enzyme was detected, serine protease S53, which expressed in GP media at least during 8 days of cultivation and whose levels increased in the presence of CuSO_4_. The protease was not detectable in LC media, likely, because of the inhibition by the products of lignocellulose degradation. In contrast, in LC media appeared an aspartic peptidase, which belongs to family of proteases with different substrate specificity, as these enzymes tend to cleave dipeptide bonds that have hydrophobic residues as well as a beta-methylene group. The existing data suggest that proteases expression is tightly regulated, so different proteases are probably expressed in different developmental stages and growth conditions. As it was demonstrated, fungal proteases are capable and likely to work in a wide range of pH [69]. At this time, no specific substrates can be suggested for both proteases.

The role of proteases in the pools of fungal extracellular proteins could be diverse. Although it was reported that LiP undergoes a protease-mediated degradation in liquid cultures of *Phanerochaete chrysosporium* [70], it is uncertain if extracellular peroxidases are substantially degraded under ligninolytic culture conditions or in colonized wood [71]. However, it was demonstrated that white rot fungi can produce significant activation of extracellular cellulases [72]; also, proteases can cleave functional domains of cellobiose dehydrogenases [73–75].

It was suggested that an aspartic protease from secretome of a plant pathogenic fungus *Botrytis cinerea* is one of virulence factors involved in host-tissue invasion and pathogenicity, *e.g.* by neutralization of plant defense proteins secreted in response to the fungal invasion [76, 77]. The authors speculated that unlike polysaccharides with a more fixed structure, plant proteins are very diverse and may require a diverse pool of proteases for degradation [77].

#### Production of cerato-platanins

When *T. hirsuta* was cultivated on LC, a considerable amount of cerato-platanins (CP) could be identified in the secretome (Fig. 3, Table 1). CPs are low molecular weight proteins with expansin-like activity on cellulose materials due to disruption of non-covalent bonds of cell wall polysaccharides but without hydrolytic activity [78]. CPs are able to cause fragmentation of the crystalline cellulose and breakage and defibration of cotton fibers [78]. Also, some of these proteins can be essential for fungal infections [79]. Genes of CPs were identified in multiple fungal genomes [80], also some information about properties of these proteins is available [40, 81]. According to localization of CPs in fungal cell, there should be at least two roles of CPs in fungi: the first one (generally referred to as the primary role) is involvement in growth and development, and the second one is the role that explains their secretion and the interaction with plants [82–84]. The data from this study indicate that CPs can be involved in the destruction of the polymer structure of plant cell wall, as they were specifically induced during the growth of *T. hirsuta* in the media with lignocellulose. It is also important that they are actively expressed at the beginning of the cultivation, suggesting that the early products of the substrate degradation are responsible for their induction and thus can activate further decay of the plant cell walls.

## Conclusions

This study of extracellular protein expression during cultivation of white rot basidiomycete *T. hirsuta* st. 072 has demonstrated the distinct composition of its secretome. The response of the fungal secretion systems on various natural inducers that appear during the fungal decay of a lignocellulose substrate, as well as on stress generating ions of copper was observed. Using MALDI-TOF/TOF MS analysis of the proteins resolved by 2D electrophoresis on different stages of the fungal growth together with monitoring enzymatic activities of lignin modifying oxidases, it was shown that during the degradation of oat straw lignocellulose the fungus secrets a distinct set of glycoside hydrolases with substrate specificity that differs from that of previously described basidiomycetes and a set of lignin modifying enzymes, in which multiple isoforms of laccases and versatile peroxidase play a major role. Also, it was determined that transfer of the fungus to lignocellulose-containing media not only induces a wide spectrum of new enzymes, but also efficiently switches the expression of other enzymes that are active in different media, thus providing a regulation of enzymatic activities of the secreted proteins. At the same time copper ions dramatically increased the activity of secreted laccases and aryl-alcohol oxidase and apparently have a mildly toxic effect on the production of other proteins in the secretome.

Thus, the data are in general agreement with previous observations on white rot basidiomycetes: the oxidoreductases are the most prominent group among the secreted enzymes, but the detection of MnPs and LiPs is less frequent in *T. hirsuta* compared to other studied basidiomycetes, with the exception of cultures grown in media developed to induce their production (see [6] for a comprehensive review). Moreover, *T. hirsuta* did not produce a significant amount of GHs and peptidases, considered the principal groups of white rot fungi secreted proteins [6]. It was also found that the fungus secreted significant amounts of cerato-platanins in response to contact with lignocellulose, indicating importance of these proteins. It is possible that the cerato-platanins assist fungal growth on natural substrates, which may include their suggested disruptive role in degradation of the lignocellulose substrates [82] and induction of CP by products of lignocellulose degradation. Therefore, the results of present research provided progress in understanding of the mechanisms of lignocellulose decay by extracellular proteins of white rot basidiomycetes. However, this in an area that needs further research.

## Abbreviations

AAO, aryl-alcohol oxidase; CBH, Cellobiohydrolase; CDH, cellobiose dehydrogenase; CP, cerato-platanin; GH, glycoside hydrolase; GP, Glucose Peptone medium; LC, lignocellulose containing medium; LiP, lignin peroxidase; LMEs, lignin-modifying enzymes; LMS, lignin-modifying system; MALDI-TOF/TOF MS, Matrix-Assisted Laser Desorption Ionization-Time-of-Flight/Time-of-Flight Mass Spectrometry; MnP, manganese peroxidase; VP, versatile peroxidase

## Additional files

Additional file 1: Optimized protocol for 2DE of extracellular proteins. (PDF 91 kb) Additional file 2: Table S2. Identification of proteins found in secretome of *T. hirsuta* st 072 by MALDI-TOF/TOF MS analysis after 2DE resolution. (PDF 110 kb)
