# Supplementary material for: Extracellular proteins of Trametes hirsuta st. 072 induced by copper ions and a lignocellulose substrate
Source: BMC Microbiol. 2016 Jun 13;16:106. doi: 10.1186/s12866-016-0729-0 (PMC4906887; doi:10.1186/s12866-016-0729-0)
Supplement: Additional file 1: — Optimized protocol for 2DE of extracellular proteins. (PDF 91 kb) [file 12866_2016_729_MOESM1_ESM.pdf]

Optimized protocol for 2DE of extracellular proteins

The quality of protein extraction and separation is the crucial part of successful identification of the proteins in proteomes. As shown earlier [1S,2S], to get sufficient quality of 2D electrophoresis for protein mapping and proteomic studies of fungi, individual procedures for each specific case need to be developed. Among the most important obstacles for the analysis are low protein concentration in the culture broth and high levels and complexity of low molecular weight components in the media and fungal metabolites. The analysis is especially difficult if cultivation is carried out on natural substrates and causes enhanced pigmentation, or at high salt concentrations. There were several attempts to develop a general approach to protein extraction from fungal liquid cultures [1S,3S,4S]; however, the successful procedures had to be adjusted individually for each organism depending on the growth conditions.

For analysis of *T. hirsuta* secretomes several procedures of protein sample preparation were tested, for which high efficiency was reported with other fungi:

i) chloroform/methanol precipitation [5S],

ii) chloroform/methanol precipitation with preliminary dialysis [6S],

iii) acetone precipitation [1S],

iv)TCA/Acetone precipitation [4S], and

v) precipitation with a commercial *2D Protein Kit* (Bio-Rad, USA) [1S].

The quality of the procedures was assessed by visualization of protein components on 2D-gels and estimation of number and resolution of individual proteins (Table 1s). Both chloroform/methanol methods did not provide sufficient protein extraction for obtaining quality protein maps even after the additional sample concentration step. Also, the acetone treatment resulted only in about 30% recovery of the proteins after precipitation, presumably, because of

partial oxidation of sulfhydryl groups, and the resulting gels contained badly resolved protein spots. For these reasons, any subsequent reprecipitation, *e.g.*, with *2D Protein Kit*, was not feasible. *2D Protein Kit* alone produced 2D gels with good resolution and quality of the protein spots; however, it did not bring about sufficient number of proteins, as it was not efficient for recovery of proteins at low concentrations. Thus, it was not suitable for the study of fungal secretomes because of low secreted proteins concentration under studied conditions, even after concentration by 10-20 fold.

In contrast, TCA/acetone precipitation was reportedly a successful method for samples with low protein concentrations and could also remove carbohydrates typical for fungal culture supernatants [4S,7S,8S]. The combination of protein precipitation with 2D Protein Kit and subsequent solubilization with lysis buffer showed the best results. Sequential protein reprecipitation provided well-resolved protein maps even for heavily pigmented LC media with a high content of fungal metabolites. As a result, 50-100 well-resolved protein components on a 2D gel were obtained, as compared to 20-200 proteins (depending on cultural media and extraction procedures) [6S,9S] usually separated in cases of other white rot fungi.

The procedure was shown to be efficient for the media used in this study. It also allowed to concentrate extracellular proteins and to remove the impeding compounds from the samples even at intense pigmentation of cultural broth. At the same time, it provided minimal loss of the proteins during the initial ultrafiltration step, as the level of total protein in the flow through the used membrane was below the sensitivity limit of the Bradford protein assay. Therefore, we used the optimized protocol, which included precipitation with a TCA/Acetone mixture followed by reprecipitation with the *2D Protein Kit* in our secretome study.

Table 1s. Comparison of extracellular proteins extraction methods for *T. hirsuta* 072

	Protein extraction	Number of protein components on gels, % of max recovered	Features of method
1	*Chloroform/methanol precipitation*	Up to 25%	Low protein yield
2	*Modified Chloroform/methanol precipitation*	Up to 25%	Low protein yield<br>Pigments contamination
3	*Acetone precipitation*	Few individual proteins	Medium protein yield<br>Poor protein recovery<br>Low resolution
4	*2D Protein Kit*	Few individual proteins	Low protein yield<br>Good resolution (no spots striking)
5	*Acetone-TCA with 2D Protein Kit*	100%	High protein yield<br>Good resolution

References

1S. Fragner D, Zomorrodi M, Kües U, Majcherczyk A. Optimized protocol for the 2-DE of extracellular proteins from higher basidiomycetes inhabiting lignocellulose. Electrophoresis. 2009;30:2431–41.

2S. Shimizu M, Wariishi H. Development of a sample preparation method for fungal proteomics. FEMS Microbiol. Lett. 2005;247:17–22.

3S. Herbert BR, Grinyer J, McCarthy JT, Isaacs M, Harry EJ, Nevalainen H, et al. Improved 2-DE of microorganisms after acidic extraction. Electrophoresis. 2006;27:1630–40.

4S. Kniemeyer O, Lessing F, Scheibner O, Hertweck C, Brakhage A a. Optimisation of a 2-D gel electrophoresis protocol for the human-pathogenic fungus Aspergillus fumigatus. Curr. Genet. 2006;49:178–89.

5S. Wessel D, Flügge UI. A method for the quantitative recovery of protein in dilute solution in the presence of detergents and lipids. Anal. Biochem. 1984;138:141–3.

6S. Zorn H, Peters T, Nimtz M, Berger RG. The secretome of Pleurotus sapidus. Proteomics. 2005;5:4832–8.

7S. Wildgruber R, Reil G, Drews O, Parlar H, Görg A. Web-based two-dimensional database of Saccharomyces cerevisiae proteins using immobilized pH gradients from pH 6 to pH 12 and matrix-assisted laser desorption/ionization-time of flight mass spectrometry. Proteomics. 2002;2:727–32.

8S. Damerval C, De Vienne D, Zivy M, Thiellement H. Technical improvements in two-dimensional electrophoresis increase the level of genetic variation detected in wheat-seedling proteins. Electrophoresis. 1986;7:52–4.

9S. Vincent D, Kohler A, Claverol S, Solier E, Joets J, Gibon J, et al. Secretome of the free-living mycelium from the ectomycorrhizal basidiomycete laccaria bicolor. J. Proteome Res. 2012;11:157–71.
