# Supplementary material for: Extracellular proteins of Trametes hirsuta st. 072 induced by copper ions and a lignocellulose substrate
Source: BMC Microbiol. 2016 Jun 13;16:106. doi: 10.1186/s12866-016-0729-0 (PMC4906887; doi:10.1186/s12866-016-0729-0)
Supplement: Additional file 2: Table S2. — Identification of proteins found in secretome of T. hirsuta st 072 by MALDI-TOF/TOF MS analysis after 2DE resolution. (PDF 110 kb) [file 12866_2016_729_MOESM2_ESM.pdf]

Table 2s. Identification of proteins found in secretome of *T. hirsuta* st 072 by MALDI-TOF/TOF MS analysis after 2DE resolution

Medium	Sequences of tryptic peptides	Suggested Enzyme	Score[5]	E-value[3]	Reference enzyme [organism]
GP	AVGPVADLTITDAAVSPDGFSRQ<br>KAVNPVPQAWSDLCPTYDALDPNDQ<br>MSRFQSLLTFINISLVAVAHAAVGPVADLTITDAAVSPDGFSRQ<br>RANPNFGNVGFDGGINSAILRY<br>RFDTNNPGPWFLHCHIDFHLEGGFAVVMAEDTPDVKA<br>RFPLGADATLINGKGRA<br>RGPFVVYDPNDPHASRYDVDNDDTVITLADWYHTAAKL<br>RSAGSTVYNYDNPIFRD<br>RYDGAPAVEPTTNQTTSVKPLNEVDLHPLVSTPVPGSPSSGGVDKA<br>RYDVDNDDTVITLADWYHTAAKL<br>RYSFVLDANQAVDNYWIRA	Laccase A[2]	235	2,E-20<br>5,E-25<br>2,E-40<br>1,E-20<br>3,E-25<br>9,E-15<br>6,E-39<br>3,E-16<br>1,E-43<br>6,E-23<br>6,E-19	AIZ72721 laccase A [Trametes hirsuta]<br>AIZ72721 laccase A [Trametes hirsuta]<br>AIZ72721 laccase A [Trametes hirsuta]<br>AIZ72721 laccase A [Trametes hirsuta]<br>AIZ72721 laccase A [Trametes hirsuta]<br>AIZ72721 laccase A [Trametes hirsuta]<br>AIZ72721 laccase A [Trametes hirsuta]<br>AIZ72721 laccase A [Trametes hirsuta]<br>AIZ72721 laccase A [Trametes hirsuta]<br>AIZ72721 laccase A [Trametes hirsuta]<br>AIZ72721 laccase A [Trametes hirsuta]
LC	AVGPVADLTITDAAVSPDGFSRQ<br>KAVNPVPQAWSDLCPTYDALDPNDQ<br>RANPNFGNVGFDGGINSAILRY<br>RFDTNNPGPWFLHCHIDFHLEGGFAVVMAEDTPDVKA<br>RFPLGADATLINGKGRA<br>RGPFVVYDPNDPHASRYDVDNDDTVITLADWYHTAAKL<br>RSAGSTVYNYDNPIFRD<br>RYDVDNDDTVITLADWYHTAAKL<br>RYSFVLDANQAVDNYWIRA	Laccase A[2]	193	2,E-20<br>5,E-25<br>1,E-20<br>3,E-25<br>9,E-15<br>6,E-39<br>3,E-16<br>6,E-23<br>6,E-19	AIZ72721 laccase A [Trametes hirsuta]<br>AIZ72721 laccase A [Trametes hirsuta]<br>AIZ72721 laccase A [Trametes hirsuta]<br>AIZ72721 laccase A [Trametes hirsuta]<br>AIZ72721 laccase A [Trametes hirsuta]<br>AIZ72721 laccase A [Trametes hirsuta]<br>AIZ72721 laccase A [Trametes hirsuta]<br>AIZ72721 laccase A [Trametes hirsuta]<br>AIZ72721 laccase A [Trametes hirsuta]
LC	RSAGSSVYNYDNPVRR<br>RYRFRLVSLSCDPNHTFSIDGHNMTIIEVDAVNHEALTVDSIQIFAAQRY	Laccase C[2]	116	1,E-14<br>2,E-51	AIZ72722 laccase C [Trametes hirsuta]<br>AIZ72722 laccase C [Trametes hirsuta]
GP	RGTLFPGTGGNQGEVESPLHGEIRLQSDSELARD<br>KTVPEPFDTVDSILARF<br>RLQSDSELARD<br>RLDVFVGRKD<br>RTACEWQSFVNNQAKLQSAFKAA	Manganese peroxidase	237	3,E-32<br>6,E-16<br>2,E-09<br>9,E-09<br>3,E-22	AFC37493 manganese peroxidase 2 [Lenzites gibbosa]<br>AFC37493 manganese peroxidase 2 [Lenzites gibbosa]<br>AFC37493 manganese peroxidase 2 [Lenzites gibbosa]<br>AFC37493 manganese peroxidase 2 [Lenzites gibbosa]<br>EIW55302 manganese-dependent peroxidase [Trametes versicolor FP-101664 SS1]
LC	RTACEWQSFVNNQAKL	Manganese peroxidase	135	1,E-15	EIW55302 manganese-dependent peroxidase [Trametes versicolor FP-101664 SS1]
GP	RLQSDFLIARD<br>RTSCEWQKM	Versatile peroxidase-like	79	8,E-10<br>3,E-09	EJF60381 manganese-repressed peroxidase [Dichomitus squalens LYAD-421 SS1]<br>EIW62513 manganese-repressed peroxidase [Trametes versicolor FP-101664 SS1]
LC	RTSCEWQKM	Versatile peroxidase-like	88	3,E-09	EIW62513 manganese-repressed peroxidase [Trametes versicolor FP-101664 SS1]
GP	RMGDQTFLGPGKT<br>KYGTGYCDTQCPHDIKF<br>RYGGICDKDGCDFNSWRM<br>KDGCDFNSWRMGDQTFLGPGKT	Cellobiohydrolase I	249	9,E-12<br>3,E-17<br>6,E-19<br>5,E-22	EIW64126 cellobiohydrolaseI [Trametes versicolor FP-101664 SS1]<br>EIW64126 cellobiohydrolaseI [Trametes versicolor FP-101664 SS1]<br>EIW64126 cellobiohydrolaseI [Trametes versicolor FP-101664 SS1]<br>EIW64126 cellobiohydrolaseI [Trametes versicolor FP-101664 SS1]
LC	RMGDQTFLGPGKT<br>KFVTHGQYSTNIGSRV	Cellobiohydrolase I	128	9,E-12<br>1,E-14	EIW64126 cellobiohydrolaseI [Trametes versicolor FP-101664 SS1]<br>EIW64126 cellobiohydrolaseI [Trametes versicolor FP-101664 SS1]

	KYGTGYCDTQCPHDIKF<br>RYGGICDKDGCDFNSWRM<br>KDGCDFNSWRMGDQTFLGPGKT			3,E-17<br>6,E-19<br>5,E-22	EIW64126 cellobiohydrolaseI [Trametes versicolor FP-101664 SS1]<br>EIW64126 cellobiohydrolaseI [Trametes versicolor FP-101664 SS1]<br>EIW64126 cellobiohydrolaseI [Trametes versicolor FP-101664 SS1]
GP	LEISDMIFSTR[1]<br>DGVTDDT[1]	Exo-β-1,3-glucanase	92	6,E-10<br>1,E-05	EIW63632exo-beta-1,3-glucanase [Trametes versicolor FP-101664 SS1]<br>AFV07042 exo-beta-1,3-glucanase variant 2 [Volvariella volvacea]
LC	QTDSHTGDGFLK [1,4]<br>QYTTHVTV[1]	Endo-β-1,3-glucanase	83	7,E-09<br>4,E-07	EIW53084 laminarinase [Trametes versicolor FP-101664 SS1]<br>EIW53084 laminarinase [Trametes versicolor FP-101664 SS1]
LC	RAFYTLGTNIIPFDPFYARM<br>RWVLESAVKS	Glycoside hydrolase family 2 protein	102	5,E-20<br>2,E-08	EIW63844 glycoside hydrolase [Trametes versicolor FP-101664 SS1]<br>EIW63844 glycoside hydrolase [Trametes versicolor FP-101664 SS1]
GP	RLAQYANDLLSLGVDGLRL	Glycoside hydrolase family 13 (α-amylase)	109	6,E-17	ETW76368 glycoside hydrolase family 13 protein [Heterobasidion irregulare TC 32-1]
LC	RFPGGNNLEGQTTATRW<br>ALPNSLQF[1]	Glycoside hydrolase family 51 protein	168	6,E-16<br>2,E-06	EIW55650glycoside hydrolase family 51 protein [Trametes versicolor FP-101664 SS1]<br>EIW55650 glycoside hydrolase family 51 protein [Trametes versicolor FP-101664 SS1]
LC	RGATAKQWMQGGS<br>RGLVDGIQEYTG<br>RTLYPLMSLHDPENFARI<br>RNLYPLMSLHDPVNFARIVRGMINIQQHEGWLPECRG	Glycoside hydrolase family 92 protein	106	2,E-11<br>3,E-10<br>1,E-17<br>4,E-39	EIW52127 glycoside hydrolase family 92 protein [Trametes versicolor FP-101664 SS1]<br>EIW52127 glycoside hydrolase family 92 protein [Trametes versicolor FP-101664 SS1]<br>EIW52127 glycoside hydrolase family 92 protein [Trametes versicolor FP-101664 SS1]<br>EGO04001 glycoside hydrolase family 92 protein [Serpula lacrymans var. lacrymans S7.3]
GP	KGSNIVFQRN<br>KAGPITNFSGIKN<br>RVAVNCGSGSCTGTWDWSALKV	Endo-polygalacturonase PG1	188	9,E-09<br>3,E-11<br>3,E-21	EIW53289 endo-polygalacturonase PG1 [Trametes versicolor FP-101664 SS1]<br>EIW53289 endo-polygalacturonase PG1 [Trametes versicolor FP-101664 SS1]<br>EIW53289 endo-polygalacturonase PG1 [Trametes versicolor FP-101664 SS1]
LC	KGSNIVFQRN<br>KAGPITNFSGIKN<br>RVAVNCGSGSCTGTWDWSALKV	Endo-polygalacturonase PG1	346	9,E-09<br>3,E-11<br>3,E-21	EIW53289 endo-polygalacturonase PG1 [Trametes versicolor FP-101664 SS1]<br>EIW53289 endo-polygalacturonase PG1 [Trametes versicolor FP-101664 SS1]<br>EIW53289 endo-polygalacturonase PG1 [Trametes versicolor FP-101664 SS1]
LC	WVENIADDIGATL[1]<br>MADYVEEV[1]	Carbohydrate esterase family 16 protein	86	3,E-12<br>6,E-08	KIJ14047 carbohydrate esterase family 16 protein [Paxillus involutus ATCC 200175]<br>KDQ32650 carbohydrate esterase family 16 protein [Pleurotus ostreatus PC15]
LC	KAGVLANIGPSGSKS<br>KFNIDETAFTDAWGRPQR	Glucoamylase (G2)	74	8,E-12<br>1,E-17	EIW63814 glucoamylase [Trametes versicolor FP-101664 SS1]<br>EIW63814 glucoamylase [Trametes versicolor FP-101664 SS1]
GP	KTLPNMPGSVTSFLAGRT<br>RQLVVGGWSLDSTFGVRL[4]	Copper radical oxidase (Glyoxal oxidase)	123	8,E-16<br>6,E-14	CDO70163 Copper radical oxidase [Trametes cinnabarina]<br>EAU83456 Copper radical oxidase [Coprinopsis cinerea okayama7#130]

GP	RAGWDPVTGLGTPNFAKL<br>LCNAYAQLGARG[1]	Family S53 protease	107	1,E-16<br>3,E-10	EIW62828 family S53 protease [Trametes versicolor FP-101664 SS1]<br>EIW62828 family S53 protease [Trametes versicolor FP-101664 SS1]
LC	KFTGSINFA<br>RATGAVFDNATGLLRI<br>RTSPASEFWGINQSIR<br>RLTQAQFAKL<br>RFYAVFDTANSR	Aspartic peptidase	238	2,E-07<br>6,E-14<br>3,E-15<br>2,E-08<br>5,E-11	EIW53588 acid protease [Trametes versicolor FP-101664 SS1]<br>EIW53588 acid protease [Trametes versicolor FP-101664 SS1]<br>EIW53588 acid protease [Trametes versicolor FP-101664 SS1]<br>EJF59364 aspartic peptidase A1 [Dichomitus squalens LYAD-421 SS1]<br>EJF59364 aspartic peptidase A1 [Dichomitus squalens LYAD-421 SS1]
LC	GFSTFGSLPHF[1]<br>DHTDAGFNIALGAMNTLTNNQ [1,4]	Cerato-platanins	112	3,E-10<br>3,E-19	EIW62257 Cerato-platanin [Trametes versicolor FP-101664 SS1]<br>EIW62257Cerato-platanin [Trametes versicolor FP-101664 SS1]

[1]Partial sequence by MSMS

[2]Nucleotide sequence of the gene for *T. Hirsuta* 072 is available

[3]Obtained by BLASTP 2.3.0 with scoring matrix PAM30 for a peptide sequence used for identification vs suggested enzyme.

[4]One amino acid replacement

[5]Best scores of initial MASCOT identification
